# Methods and Strategies for Reconditioning Motor Output and Postural Balance in Frail Older Subjects Prone to Falls

**DOI:** 10.3389/fphys.2021.700723

**Published:** 2021-10-12

**Authors:** Thierry Paillard

**Affiliations:** Laboratoire Mouvement, Equilibre, Performance et Santé (UPRES EA 4445), Département STAPS, Université de Pau et des Pays de l’Adour/E2S, Pau, France

**Keywords:** balance, postural control, postural balance, muscle strength, muscle power, fall, elderly, older

## Abstract

In frail older subjects, the motor output of the antigravity muscles is fundamental in resisting falls. These muscles undergo accelerated involutions when they are inactive and the risk of falling increases during leisure and domestic physical activity. In order to reduce their risk of falling, frail older subjects limit their physical activities/exercises. The problem is that the less they exercise, the less they are able to exercise and the greater the risk in exercising. Hence, a vicious circle sets up and the antigravity muscles inevitably continue to deteriorate. This vicious circle must be broken by starting a reconditioning program based on developing the strength of antigravity muscles (especially lower-limb muscles). To begin with, for each increase in muscle strength, postural balance is improved. Once this increase reaches the threshold beyond which postural balance no longer improves, it seems appropriate to implement exercises aimed at concomitantly improving motor output and postural balance in order to counteract or even reverse the involution process of the postural balance system. Methods and strategies toward this end are proposed in this present communication. However, the transfer effects between strength increase and postural balance ability are not yet totally known and future research should evaluate the relationship between muscle strength and postural balance throughout rehabilitation programs (i.e., program follow-ups) in frail older subjects in order to advance knowledge of this relationship.

## Introduction

Advancing age can be characterized by structural and functional alterations of the organism that provoke disturbances of the regulatory mechanisms, which generate a reduction of the adaptation capacities in maximal/intense activities and also in the activities of the daily life. Gradually, over the years, this process makes more and more activities (including domestic activities) difficult or even impossible for very old and frail persons. Certain activities can even potentially endanger/expose their physical integrity. In fact, the simple act of walking may involve real risks of falling with all the health, social, and economic complications that this may entail: for example, bone fracture, trauma, immobilization, institutionalization, and hospitalization (Sanders et al., 2017).

In order to reduce the risk of falling, frail older adults voluntarily limit their domestic physical activities (Matsuda et al., 2020). The problem is that the less they exercise, the less they are able to exercise and the more risk they take in exercising. Hence, a vicious circle sets up and the antigravity muscles inevitably continue to deteriorate. The ultimate result of this process is that frail older adults lose their physical independence. In order to break this vicious circle, it is necessary to exercise before the ability to move disappears completely. Indeed, only regular physical activity stimulates the basal motor skills needed for activities of the daily life such as, for instance, to stand up, walk, sit, pick up, and carry objects.

The performance of all these basal motor tasks implies the ability to regulate balance and movement on the basis of efficient sensory (proprioceptive, cutaneous, vestibular, and visual cues), central (command and control of movement), and motor (motor execution) functions (Paillard, 2017a). Evidence suggests that fall risk in the frail elderly involves multifactorial and systemic dimensions (sensory, cognitive, and motor pathways; Maidan et al., 2020). By regularly activating these functions through physical activity, their alteration with advancing age is reduced, even if they continue to be altered in a more limited way (Perrin et al., 1999). Hence, when individuals are close to losing their ability to move, there is an urgent need to exercise. However, in frail older adults, exercise is a source of imbalance since the simple fact of walking constitutes a succession of transient imbalances (provoked by the successive alternation of monopodal and bipodal supports) that must be instantly restored to prevent a fall (van Schooten et al., 2018).

There is a dilemma since exercise is required to stimulate sensory, central, and motor functions in order to preserve the ability to move but it is also potentially source of falls and induced injuries (Stathokostas et al., 2013). As long as postural balance (which can be defined as the ability to control the center of gravity to prevent the body from falling) remains above a certain level of control (providing a minimum of safety in the execution of the movement in order to limit the risk of falling), stimulation of the different sensory, central, and motor functions can be carried out successively or simultaneously by adapted physical activities (e.g., balance, strength, flexibility, and endurance exercises; Perrin et al., 1999; Paillard, 2017b). By contrast, below this level, there is a high risk of loss of balance due to imbalances caused by physical activity, and particular strategies must be deployed to improve the ability of frail older subjects to move safely. These strategies are primarily based on the enhancement of the motor function since, before starting regular physical activities involving body movement in frail older subjects prone to falls, it is necessary to augment their motor output to facilitate movement in complete safety regardless the control of the center of gravity. This improved motor function should be a priority in frail older subjects prone to falls. This hypothesis is based on the fact that it has already been shown in physically diminished old subjects with knee osteoarthrisis that there is a relationship between domestic tasks such as stair-climbing ability and the maximal knee extensor strength while there is no relationship between stair-climbing ability and postural balance (Casaña et al., 2021).

Hence, the aim of this communication is to propose methods and strategies for improving (reconditioning) the ability to move safely in frail older subjects prone to falls. They are based, first, on the analysis of the contextual impact of motor output (muscle strength and power) of antigravity muscles (i.e., muscle chains that resist falls) on postural balance and, second, on the design of progressive adapted exercises.

## Fundamental Role of Antigravity Muscles

From a motor viewpoint, postural balance is mainly achieved by the extensor muscles of the head, trunk, thigh, leg, and foot (Schieppati et al., 2003; Cattagni et al., 2014; Paillard, 2017b; Golubić et al., 2019; Quinlan et al., 2020). The motor output of most of these antigravity muscles has often been tested in association with postural balance. Low and very low output of these muscles would affect postural balance negatively in older adults (Horlings et al., 2008; Forte et al., 2014; Paillard, 2017b). In this context, a low motor output of extensor muscles would disturb postural balance more than that of the flexor muscles. For example, loss of strength of the plantar flexor muscles would be more disruptive than that of the dorsiflexor muscles on postural balance particularly in the sagittal plane (Melzer et al., 2009). Moreover, weakness in both distal and proximal extensor muscles (on the whole body) leads to disturb postural balance in subjects over 50years of age (Horlings et al., 2009).

At the lower-limb level, there would be a relationship between ankle or knee extensor muscles strength and postural balance in healthy and pathological (e.g., cerebral palsy or stroke patients) older subjects (Horlings et al., 2008; Melzer et al., 2009; Orr, 2010; Forte et al., 2014; Gomes et al., 2015; Han and Yang, 2015; Svoboda et al., 2019; Jensen et al., 2020; Mentiplay et al., 2020; Uysal et al., 2020; Yoshizawa et al., 2020; Casaña et al., 2021; Tavakkoli Oskouei et al., 2021). The level of strength produced by these muscles may reduce or amplify the risk of falling in older subjects (Horlings et al., 2008; Scott et al., 2014). It has been suggested that there would be a threshold below which the lack of muscle strength results in deterioration of postural balance (Paillard, 2017b). As long as this threshold is not reached, the slightest increase in muscle strength would be sufficient to improve postural balance (Paillard, 2017b) and would be likely to reduce the risk of falling (Scott et al., 2014). In turn, when the level of strength is beyond this threshold, an increase in lower-extremity muscle strength cannot improve postural balance even after training programs while a decrease in muscle strength would not accompanied by a degradation of postural balance (Orr et al., 2008; Howe et al., 2011; Granacher et al., 2012; Muehlbauer et al., 2012; Sculthorpe et al., 2017).

At the trunk level, there would also be a relationship between trunk extensor muscle strength and power and postural balance ability in older subjects (Golubić et al., 2019; Taveira et al., 2021). The thickness of the trunk muscles may be associated with postural balance, as older adults with thicker trunk muscles have better postural balance (Acar et al., 2020). At the foot level, intrinsic muscles strength would also influence postural balance. The systematic review by Quinlan et al. (2020) showed that strengthening toe flexor muscles would improve postural balance in adults aged 60years and older. The level of strength of the toe muscles would be particularly influential since asymmetry in toe grip strength causes a disturbance in postural balance (Koda et al., 2018). In addition, increases in hallux grip force (hallux being the fundamental toe for regulating postural balance) would be associated with improved postural balance (Chatzistergos et al., 2019). These authors reported that hallux grip force would be correlated to the strength of all muscle groups of the foot-ankle complex.

Overall, extensor/antigravity muscle strength measurements could potentially assess the fall risk in frail older adults. Valenzuela et al. (2020) recently detected differences between fallers and non-fallers among older adults on the basis of simple measurements of maximal voluntary isometric strength of the knee extensors even when adjusting for potential confounding factors, such as age, gender, body mass index, and previous history of falls. Measurements to detect sarcopenia (expressed as muscle mass/height^2^) could also help predict postural balance disorders (Kim et al., 2020). Based on the results mentioned above, it is worth considering whether force variations of these antigravity muscles have an impact on postural balance under standard and challenging postural conditions.

## Involution of Antigravity Muscles

With advancing age, the musculoskeletal and articular systems undergo structural and functional involutions that generate reductions of muscle contractility and stiffness and regression of intermuscular coordination (agonist-antagonist muscle co-contraction) as well as shortening in the range of motion of eversion-inversion of the foot (Vandervoort, 2002; Menz, 2015). All these involutions degrade the motor output and motor component of the postural balance system (Bok et al., 2013; Cattagni et al., 2014; Menz, 2015). On the basis of these motor alterations, for a given postural balance condition, the effort required to control body balance is greater in older subjects than in young subjects and needs greater electromyographic activity to produce appropriate torque (Billot et al., 2010; Nagai et al., 2011; Skurvidas et al., 2012; Donath et al., 2016). It can be assumed that below a certain level of muscle strength (ability to produce strength), the increase in electromyographic activity to compensate for the age-related reduction in motor output is no longer possible and postural balance is negatively affected (Paillard, 2017b). Regarding the plantar flexor muscles, for example, Cattagni et al. (2014) revealed that below the threshold of 3.1N·m·kg^−1^, postural balance was clearly degraded and body balance was no longer safely ensured. Overall, the effects of the involution of antigravity muscles on postural balance would be relatively quantifiable under standard evaluation conditions (static and/or stable) but they would be more difficult to measure (apprehend) under ecological evaluation conditions (dynamic and/or unstable) i.e., when time constraints are more prevalent.

## Motor Output of Antigravity Muscles in Ecological Condition

Under standard and static postural balance conditions, the relationship between lower-limb muscle strength and postural balance can be reliably established (Paillard, 2017b; Casaña et al., 2021). However, under ecologic and dynamic conditions, this relationship is no longer observed (Paillard, 2017b).

In fact, under ecological conditions, the time factor is fundamental since the slightest body imbalance needs to be counteracted as fast as possible by opposing forces to overcome the time-dependent increasing inertia of the body movements and therefore prevent the fall. It is thus no longer a question of the level of absolute muscle force (maximum voluntary contraction) independent of the time factor but rather of the level of force in relation to time. In this case, the fundamental factor would be the muscle power (force×velocity) and/or the rate of force development (RFD) in order to avoid falling. The postural balance ability is associated with the RFD relative to maximal voluntary contraction (MVC) of the plantar flexion but not with the absolute MVC (Ema et al., 2016). The variation in all center of foot pressure (COP) amplitudes (displacements) is inversely related to the RFD scaling factor (20, 40, 60, and 80%) of hip abductor (Kozinc et al., 2020). Moreover, lowering the RFD would reduce the capability to efficiently and instantaneously counterbalance imbalances that occur. Since muscle contraction speed decreases with advancing age, it is logical that the RFD is lower in older subjects, which is accompanied by a reduction of the capability to counteract incipient body imbalance through rapid postural adjustments (Izquierdo et al., 1999). This lowing of the RFD would be more important in older fallers than in non-fallers subjects (Fleming et al., 1991). Inversely, older subjects exhibiting higher RFD also display better postural balance among a sample of age-matched subjects (Sundstrup et al., 2010). The RFD would constitute a key factor to resist potential body imbalances likely to induce falls. However, efficient postural reactions involve not only high RFD to avoid falling but also very short reaction times (i.e., the delay between the imbalance signals and the beginning of the contraction producing opposed forces) because of the time-dependent increasing inertia as mentioned below. During gait, falls occur a very short time after perturbation, between 200 and 500ms (Han and Yang, 2015). In a context of postural balance evaluation, the forces required for opposing body imbalances must be produced even faster than 200ms (Ema et al., 2016). These authors clarified that the relationship between the RFD and the COP displacement exists only for 30, 50, 100, and 150ms delays but not for 200ms.

Moreover, the variability factor in force development could also turn out to be influential since there would be also a relationship between COP displacement and plantar flexor muscle force variability on an unstable platform in older adults (Hirono et al., 2021). However, the level of force should be relatively high because with low values of RFD around 5 and 10% of the plantar flexor muscle MVC (with and without the aid of visual feedback of the force produced); this relationship is no longer observed (Barbosa et al., 2018). This would mean that high postural balance abilities in older subjects would require low force variability in the postural balance regulation but high RFD values in the lower limb antigravity muscles. In order to optimize postural balance in older subjects, suitable reconditioning/training would be required to decrease the force variability (Barbosa et al., 2020) and increase the RFD of antigravity muscles.

When the motor output of antigravity muscles has previously been improved in frail older subjects, it becomes possible to specifically stimulate the sensory and central components of the postural balance system in complete safety (i.e., by limiting the fall risk) by appropriate and progressive exercises.

## Reconditioning of Antigravity Muscles

**Figure 1:**
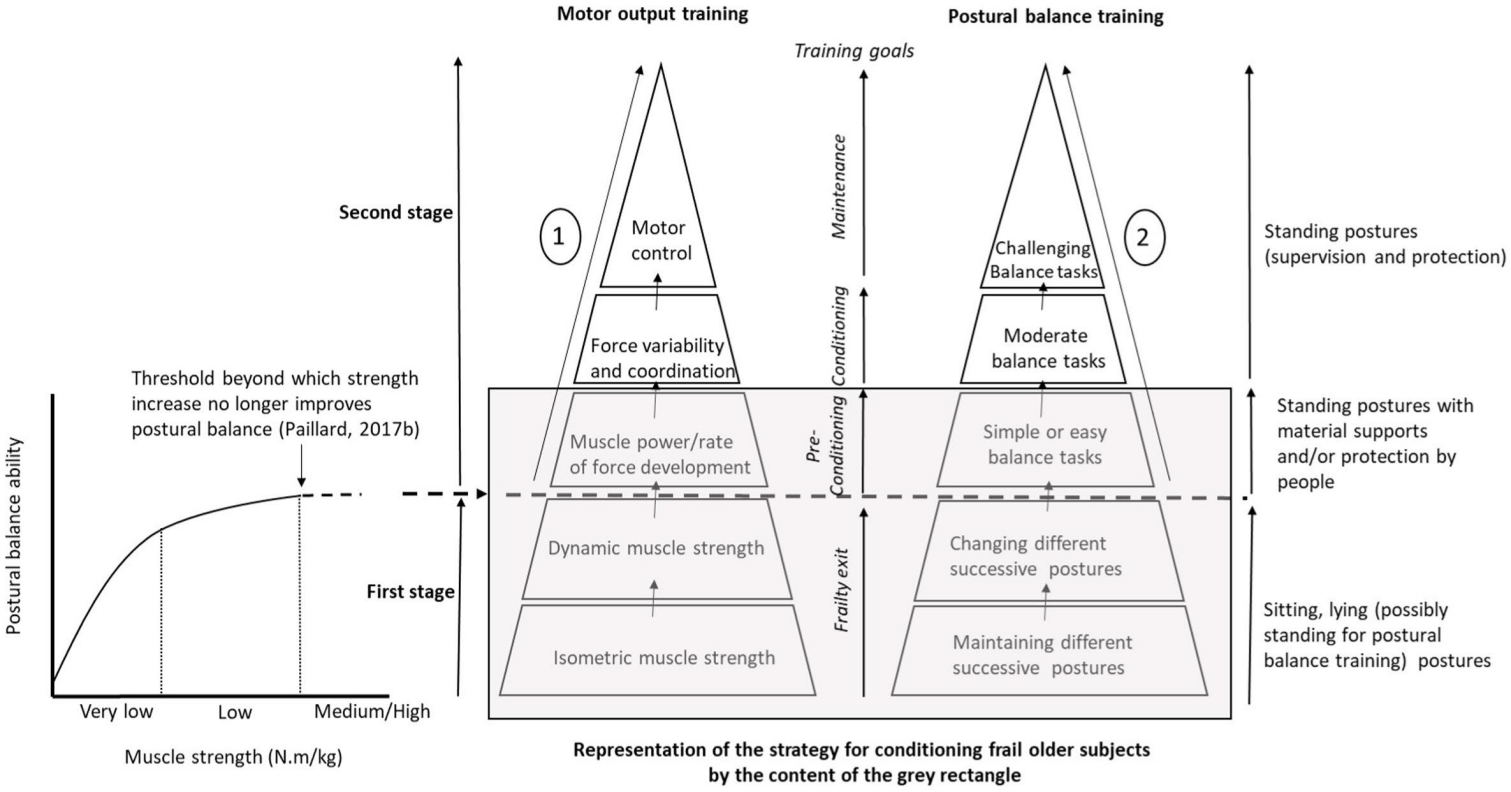
Representation of the proposed methods and strategies for reconditioning motor output (antigravity muscles) and postural balance in frail older subjects prone to falls. At first, for each increase in muscle strength, postural balance is improved-curve and threshold on the left side of the figure (First stage). Once this increase reaches the threshold beyond which postural balance is no longer improved, it seems appropriate to implement exercises aimed at concomitantly improving motor output and postural balance in order to counteract or even reverse the involution process of the postural balance system (Second stage). This is a global theoretical strategy since frail older subjects are only able to achieve the first stage and the first step of the second stage. Regarding the last steps of the second stage, older subjects must no longer be fragile in order to undertake them. Only after completing the preconditioning training can initial frail older subjects continue the proposed process. For the global theoretical strategy, only preconditioned older subjects would be able to apply it completely at the motor and postural levels. ① Motor training – Firstly, muscle power/rate of force development (RFD), coordination and movement control should be progressively developed under ecological conditions by carrying, moving, throwing, and controlling objects. Secondly, all these physiological qualities (including aerobic and flexibility qualities) should be mobilized under stimulating conditions. ② Postural training – Based on different postural positions and conditions, the relevant reconditioning in frail older subjects requires postural balance tasks from easy to difficult. These tasks include different postures (bipedal and monopedal), bases of support (static-dynamic; large-narrow; and firm-foam), shoeing condition (dress shoes, sports shoes, shoes with textured insoles, and barefoot), visual condition (eyes open, eyes closed, and visual manipulations), nature of tasks (balance task alone, combined with cognitive tasks, i.e., dual-task), temporal constraints (with or without reaction time requirements, responses to sound and visual signals), and variation of postural constraints (no disturbance, expected or unexpected disturbances). For most of postural balance tasks, a multitude of variants can be suggested related to feet position (close feet, semi-tandem, and tandem stances), body segmental position or movement (e.g., arm crossed, hip and knee flexed, different head placements, and heel or toe contact with the ground), and light and acoustic environments (very or not enough light, without any noise or with music) etc. The overall strategy (first and second stages) is based first on analytical/static tasks, then on local/dynamic tasks, and finally on global/dynamic tasks for motor and postural training.

In frail older subjects prone to falls, evidence suggests that the ability to move (i.e., the motor and postural skills which can be defined as the ability to efficiency and safely control segmental movements and the center of gravity, respectively) should be progressively reconditioned while avoiding any risk of falling. To this end, physical activity is fundamental in order to counteract and even reverse the process of involution for the ability to move with advancing age. However, in frail older subjects, the slightest postural disturbance is likely to cause a fall. It is a vicious circle that must be broken. Specific methods and strategies should be deployed to achieve this goal. Hence, the first aim is to perform exercises to improve their ability to move safely. In the first instance, exercise should be performed in a state of sustained body stability in order to simultaneously recondition muscle strength and the motor function (the whole antigravity muscles linked to the lower-limb and trunk) while strictly avoiding the risk of falling in frail older subjects. Motor and postural skills are the two factors to consider in reconditioning methods and strategies. Based on the relationship between lower-extremity muscle strength (e.g., triceps surae and quadriceps femoris) and postural balance in frail older subjects displaying low and very low lower-extremity muscle strength (Paillard, 2017b), the development of maximal muscle strength turns out to be crucial in the reconditioning process (Figure 1 – First stage). From a motor viewpoint, as long as the lower-limb maximal muscle strength is low and its increase improves postural balance, the priority is to develop it in isometric action mode (including static muscle actions) before developing it in dynamic mode (including concentric and excentric muscle actions) in a sitting or lying position (posture assured). From a postural viewpoint, maintaining and changing different successive postures (sitting and standing) should be safely accomplished and/or sustained (i.e., material supports and/or protection by people). Once this initial stage has been completed, when an increase in maximal muscle strength is not sufficient to improve postural balance, older subjects can begin new progressive tasks in order to optimize motor output (i.e., motor function) through exercises aimed at improving muscle power, RFD (standing posture with material supports and/or protection by people), force variability, coordination, and movement control (standing posture with supervision and protection; Figure 1 – Second stage). During this second stage, postural balance exercises can start with simple and easy balance tasks (e.g., under bipedal static condition with close feet, semi-tandem, and tandem stance, on slightly narrowed floor surfaces) in order to initiate stimulation of the sensory and central components of the postural balance system. Among central components, the cognitive function needs to be stimulated early through a dual task (ecological postural condition) because poor cognitive ability would be associated with poor postural balance ability in frail older subjects (Bramell-Risberg et al., 2012; Taylor et al., 2021). This need is reinforced by the fact that the cortical contribution increases during continuous postural balance tasks with advancing age (Mouthon et al., 2018; Ozdemir et al., 2018). Evidence suggests that the progress of reconditioning is limited with frail older subjects since their motor and postural basal level is very low. However, with subjects who are highly adaptable to reconditioning training, as well as older subjects who are more alert at baseline, the content of exercises can continue to increase in difficulty in order to stimulate more strongly motor and postural functions. Hence, it becomes possible to undertake other progressive steps (cf. Figure 1 and its legend – Second stage) in order to improve motor output and postural balance in this type of older subject. Overall, the last two steps of the second stage do not involve frail older subjects prone to fall, but rather older subjects who have either relatively good motor and postural abilities, or subjects who already have benefited from preconditioning training (i.e., who have completed the first stage).

## Conclusion

In frail older subjects prone to falls, the strength of antigravity muscles turns out to be essential as long as its increase permits improve postural balance. Once this increase reaches the threshold beyond which postural balance is not improved, methods and strategies related to motor output and postural balance should be progressively and safely implemented to counteract or even reverse the process of involution of the postural balance system. In practice, when the lower-limb maximal muscle strength is too low, the priority is to develop it in isometric action mode (including static muscle actions) before developing it in dynamic mode (including concentric and excentric muscle actions) in a sitting or lying position. From a postural viewpoint, the maintaining and changing different successive postures (sitting and standing) should be safely accomplished and/or sustained (i.e., material supports and/or protection by people). When increasing maximal muscle strength is not sufficient to improve postural balance, the RFD, coordination, and motor control should be targeted. From a postural viewpoint, exercises should be performed under bipedal static condition (e.g., close feet, tandem, and semi-tandem stance, on slightly narrowed floor surfaces) and should include dual tasks early in the reconditioning process since the cognitive function is not only impaired but also associated with the postural balance function in frail older subjects. Other more difficult exercises can be used, but only in older subjects who have reached a minimum level of motor and postural skills after reconditioning. However, the transfer effects between strength increase and postural balance ability are not yet totally known and future research should evaluate the relationship between muscle strength and postural balance throughout rehabilitation programs (i.e., program follow-ups) in frail older subjects in order to advance knowledge of this relationship.

## Author Contributions

The author confirms being the sole contributor of this work and has approved it for publication.

## Conflict of Interest

The author declares that the research was conducted in the absence of any commercial or financial relationships that could be construed as a potential conflict of interest.

## Publisher’s Note

All claims expressed in this article are solely those of the authors and do not necessarily represent those of their affiliated organizations, or those of the publisher, the editors and the reviewers. Any product that may be evaluated in this article, or claim that may be made by its manufacturer, is not guaranteed or endorsed by the publisher.
